# Mixed Cluster Ions of Magnesium and C_60_

**DOI:** 10.1021/acs.jpca.3c06902

**Published:** 2024-01-25

**Authors:** Anna Maria Reider, Jan Mayerhofer, Paul Martini, Paul Scheier, Olga V. Lushchikova

**Affiliations:** † Institut für Ionenphysik und Angewandte Physik, Universität Innsbruck, Technikerstr. 25, A-6020 Innsbruck, Austria; ‡ Department of Physics, Stockholm University, 106 91 Stockholm, Sweden

## Abstract

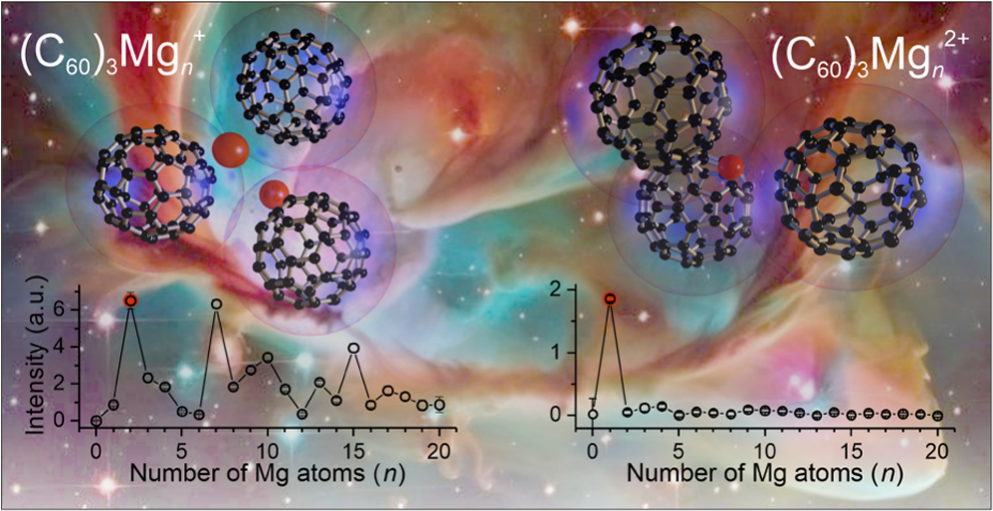

Magnesium clusters exhibit a pronounced nonmetal-to-metal transition, and the neutral dimer is exceptionally weakly bound. In the present study, we formed pristine Mg_*n*_^*z*+^ (*n* = 1–100, *z* = 1–3) clusters and mixed (C_60_)_*m*_Mg_*n*_^*z*+^ clusters (*m* = 1–7, *z* = 1, 2) upon electron irradiation of neutral helium nanodroplets doped with magnesium or a combination of C_60_ and magnesium. The mass spectra obtained for pristine magnesium cluster ions exhibit anomalies, consistent with previous reports in the literature. The anomalies observed for C_60_Mg_*n*_^+^ strongly suggest that Mg atoms tend to wet the surface of the single fullerene positioning itself above the center of a pentagonal or hexagonal face, while, for (C_60_)_*m*_Mg_*n*_^*z*+^, the preference for Mg to position itself within the dimples formed by fullerene cages becomes apparent. Besides doubly charged cluster ions, with the smallest member Mg_2_^2+^, we also observed the formation of triply charged ions Mg_*n*_^3+^ with *n* > 24. The ion efficiency curves of singly and multiply charged ions exhibit pronounced differences compared to singly charged ions at higher electron energies. These findings indicate that sequential Penning ionization is essential in the formation of doubly and triply charged ions inside doped helium nanodroplets.

## 1 Introduction

Complexes of metals and fullerenes exhibit exceptional properties that are proposed to be beneficial for various applications, including catalysis,^1,2^ biosensors,^3,4^ hydrogen storage,^5−7^ superconductivity,^8,9^ and optoelectronics.^10,11^ On the computational side, a vast amount of papers on this topic can be found in the literature,^12^ including several highly cited reviews.^13−15^ In contrast, much fewer experimental studies have been published albeit the fact that the first paper on metal–fullerene complexes was the mass spectrometric detection of lanthanum–fullerene complexes formed by laser vaporization of graphite that was exposed to a boiling saturated solution of LaCl_3_.^16^ This paper was published in 1985, within one week after the discovery of fullerenes^17^ by the same group. Based on the unusual stability of this metal–fullerene complex upon intense laser irradiation, they proposed that this tightly bound lanthanum atom is located inside the fullerene cage. It took several more years to finally confirm this hypothesis.^18^ X-ray photoelectron spectroscopy of endohedral lanthanum–fullerene complexes revealed that the lanthanum atom inside a carbon cage is triply positively charged and protected from reactions with water or oxygen.^19^ According to ab initio electronic structure calculations, the position of the metal in endohedral fullerenes is off the cage center.^20^

Robledo et al. implemented density functional theory to calculate the most stable exohedral metal–fullerene cationic complexes for the first three alkali and alkaline earth metals, as well as for the first-period transition metals.^21^ For Li, the binding energies on top of a hexagonal or pentagonal ring are almost equal, whereas for Na and K, the hexagonal position is energetically favorable by 0.54 and 1.25 eV, respectively. Be and Mg turn out to prefer to bind to two carbon atoms connecting a pentagonal and hexagonal ring, whereas the most stable Ca isomer has the metal located on top of a pentagonal ring. Most transition metals bind to two carbon atoms connecting two hexagonal rings.

In 1991, the superconductivity of potassium-doped C_60_ was discovered in both films and bulk samples.^9^ In this exohedral structure, C_60_ forms a face-centered cubic arrangement by incorporating K ions into all of the octahedral and tetrahedral interstices of the host lattice.^22^ It is interesting to note that heavily doped K_6_C_60_ has a body-centered cubic structure, with K atoms in distorted tetrahedral sites.^23^ Zimmerman and Hercules reported the formation of exohedral metal–fullerene complexes containing one metal ion and up to three fullerene units in mass spectra obtained by argon-ion bombardment of fullerenes deposited on metal surfaces.^24^

Later, the groups of Martin^25−27^ and Kaya^28^ utilized gas aggregation and mass spectrometry to examine the decoration of C_60_ with various metals. The high vapor pressure of alkali and alkaline earth metals at relatively moderate temperatures enabled the formation of C_60_M_*n*_^+^ complexes with *n* up to 500 Ca^27^ and Cs^26^ atoms. In the case of Ca, pronounced intensity anomalies at *n* = 32, 104, 236, and 448 were assigned to the closure of nested icosahedral shells,^27^ whereas the clusters ions of C_60_ heavily decorated with Cs exhibit electronic shell closures.^26^ The first shell closure of *n* = 32 corresponds to the number of faces of the C_60_ cage that consists of 12 pentagons that are isolated from each other by 20 hexagons. This commensurate decoration of the fullerene surface has been observed for several small molecular adsorbates, both experimentally^29−31^ and computationally.^32^ For lower metal coverage, the interaction between the metal and fullerene leads to various decoration patterns. Li preferentially occupies sites on top of pentagonal rings and exhibits a magic number at *n* = 12, whereas the larger alkali metals lead to the formation of C_60_M_6_ building blocks.^33^

Hou et al. measured infrared absorption spectra of C_60_Fe^+^ and C_60_V^+^ via photofragmentation of Ar- and D_2_-tagged complexes, respectively, and comparison with observational spectra from several fullerene-rich planetary nebulae exhibits a positive linear correlation.^34^ Furthermore, a follow-up study by the same group included quantum chemical calculations and molecular dynamics simulations using DFT to determine the most favorable binding site of a vanadium cation to be on top of a pentagonal ring.^35^ These findings contrast with those of Robledo et al., who found the lowest energy for a position on top of a hexagonal ring.^21^ However, within the computational uncertainties, both positions are equally stable.

An alternative method to form metal–fullerene complexes is the sequential pickup of metal atoms and fullerenes into helium nanodroplets (HNDs).^36^ Harnisch et al. formed mixed cationic clusters of C_60_ and the alkali atoms Na and Cs by electron ionization of helium droplets doped with C_60_ and one of the two alkali metals.^37^ For singly and doubly charged cations, C_60_M_6_ building blocks were observed in agreement with previous gas aggregation studies^33^ for complexes containing up to 10 fullerenes. For substantially lower doping with Cs, particularly intense clusters of the form (C_60_)_*m*_Cs_*n*_^±^ are found for *n* = 3 and to a smaller degree for *n* = 5.^38^ Enhanced hydrogen adsorption to C_60_Cs^+^ was observed compared to bare C_60_^+^ by analyzing anomalies in the ion abundance of hydrogen-covered C_60_Cs^+^ ions.^39^ Goulart et al. reported the existence of highly stable C_60_–Au–C_60_ dumbbells, both positively and negatively charged,^40^ and Martini et al. extended these studies of coinage metal–fullerene complexes (C_60_)_*m*_M_*n*_^±^ with *m* up to 10 fullerenes and *n* up to 20 metal atoms of copper and gold.^41^

In the literature, several experimental,^42−56^ as well as theoretical studies,^57−62^ can be found for magnesium-doped HNDs. In most of these studies, the HNDs were doped with magnesium only. The group of Tiggesbäumker discovered electronic shell closures for pristine Mg_*n*_^+^ clusters formed in HNDs via pronounced intensity anomalies.^43^ In subsequent studies, this group discovered that magnesium atoms embedded in HNDs arrange themselves in a metastable network, referred to as “foam”, and investigated its time-resolved^46,48^ and spontaneous^56^ collapse. The group of Lindsay studied the deposition of pristine magnesium nanoparticles^49,50,63^ as well as composite films of nanoparticles containing perfluoropolyether^51^ and films of magnesium–copper core–shell nanoparticles,^52^ all formed in HNDs. Krasnokutski and Huisken investigated the chemical reactions of Mg and O_2_ inside HNDs^47^ and the group of Miller measured rotationally resolved spectra of HCN–Mg complexes in HNDs.^42,64^

In the present study, we investigate cationic magnesium cluster ions as well as mixed cationic clusters of magnesium and C_60_, which, despite their astrophysical relevance, have not been studied before. The clusters are formed upon pickup into neutral HNDs that are ionized by multiple electron impacts and analyzed by high-resolution mass spectrometry.

## 2 Materials and Methods

All measurements were performed with a setup that was explained in detail before^65^ and slightly modified for the present needs. HNDs were formed upon expansion of precooled (9.6 K) and pressurized (2.2 MPa) high-grade He (purity 99.9999%, Messer Austria GmbH) through a pinhole nozzle (Plano GmbH, A0200P) with a diameter of 5 μm. The resulting droplets contain, on average, about 5 × 10^5^ He atoms and pass a conical skimmer (Beam Dynamics, Inc.) about 1 cm downstream from the nozzle. In two differentially pumped vacuum chambers (residual gas pressure without HNDs < 10^–6^ Pa), the droplets are doped sequentially by passing through ohmically heated ovens filled with C_60_ (SES Res. 99.99%, 516 K) and Mg (Alfa Aesar 99.98%, 632 K). C_60_ is heliophilic and will submerge into the HNDs where clusters are formed, and the Mg atoms are expected to attach to these C_60_ clusters. As in the case of Cu and Mg, the formation of core–shell structures can be expected with a fullerene core and a magnesium shell.^52^ Further downstream, the beam of doped HNDs is crossed with an electron beam with a variable electron energy (0–150 eV). At an electron current of 132 μA, the average-sized HND is hit by about ten electrons, which will lead to the formation of several He^+^ ions and electronically excited He*. Resonant hole hopping toward the dopant cluster will lead to charge transfer from He^+^, and the formation of an electronically excited cationic dopant cluster, potentially doubly charged. He* is heliophobic and unlikely to interact with submerged neutral dopant clusters due to the weak van der Waals interaction. However, ion-induced dipole interaction between ions and highly polarizable He* enables additional reactions such as Penning ionization^66^ and electronic excitation of dopant clusters inside the HNDs. Coulomb repulsion between all positively charged ions leads to the ejection of low-mass ions that are guided toward the extraction region of an orthogonal reflectron time-of-flight mass spectrometer (TOFWERK HTOF, resolving power *m*/Δ*m* = 2500). Mass spectra were taken at different temperatures of the magnesium oven at an electron energy of 70 eV and an electron energy scan was taken from 0 to 50 eV in steps of 0.1 eV. The resulting mass spectra are dominated by He_*k*_^+^ ions up to *k* = 179. At higher mass per charge values, dopant cluster ions of the form (C_60_)_*m*_Mg_*n*_^+^ are prevalent. The natural isotopes of carbon and magnesium lead to complex isotopic patterns that quickly start to overlap. The software IsotopeFit^67^ was used to extract the yield of mixed magnesium–fullerene complexes, taking into account the natural isotopic contributions of Mg and C, the peak shape of the instrument, and a potential background signal.

## 3 Results

### 3.1 Mass Spectra and Cluster Size Distributions

#### 3.1.1 Pristine Magnesium Cluster Ions

**Figure 1:**
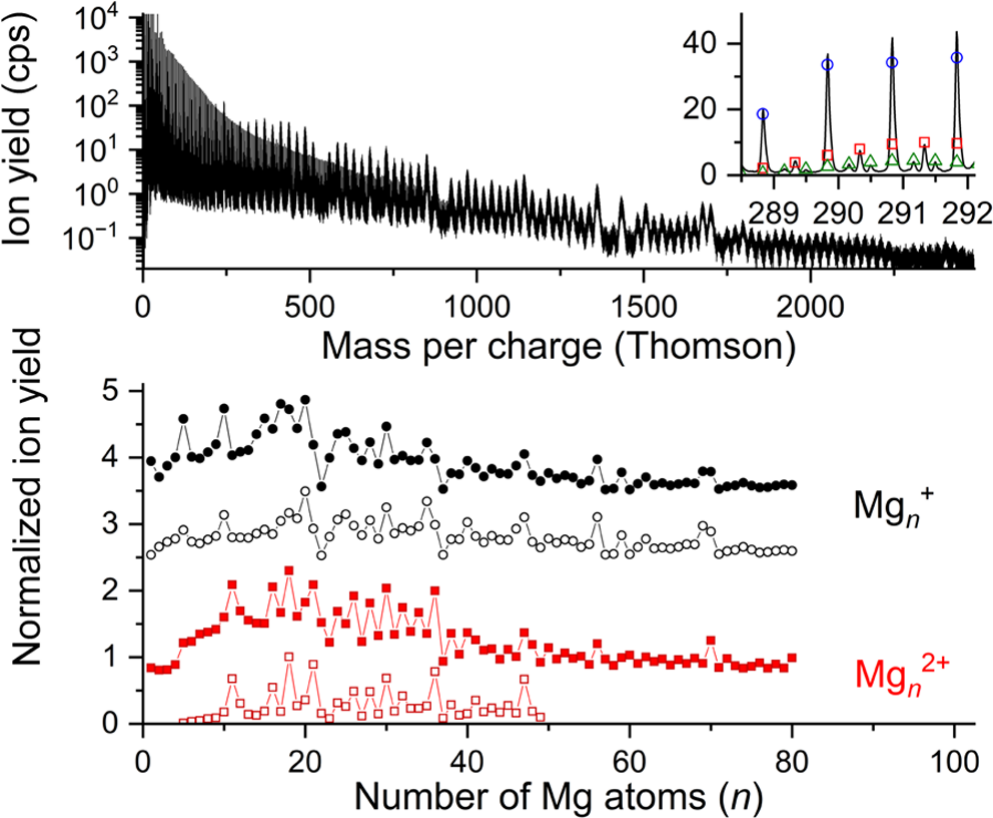
Mass spectrum of low-mass ions ejected from neutral HNDs doped with magnesium upon multiple electron bombardment (upper diagram). At mass per charge values below 240 Thomson, the mass spectrum is dominated by He_*k*_^+^ ions that exhibit an almost biexponential decrease of the ion yields as a function of *k*. At higher mass per charge values, Mg_*n*_^+^ is the prevailing ion series. Pronounced intensity anomalies can be seen in this semilogarithmic plot. The inset shows a small section of the same mass spectrum, and individual mass peaks were assigned to the isotopic patterns of Mg_12_^+^ (blue circles), Mg_24_^2+^ (red squares), and Mg_36_^3+^ (green triangles). In the lower diagram, the Mg_*n*_^+^ distribution is shown with black circles, the solid ones were deduced with the software IsotopeFit^67^ from the mass spectrum above, and the resulting cluster size distribution is compared to a Mg_*n*_^+^ cluster size distribution from the literature^43^ (open circles). The Mg_*n*_^2+^ distribution is depicted with red squares: the solid ones represent data obtained via IsotopeFit from the mass spectrum shown in the upper diagram, and the open squares are taken from Diederich et al.^68^

The upper diagram in Figure 1 shows a mass spectrum obtained by electron ionization of neutral HNDs (average size 5 × 10^5^ atoms) doped with magnesium only (the C_60_ oven is heated to 390 K where the vapor pressure of fullerenes is too low to contribute to the mass spectrum). A logarithmic scale was chosen for the *y*-axis since pristine helium cluster ions He_*k*_^+^ are by far the most abundant ions. The first magnesium cluster that exceeds the He_*k*_^+^ series is Mg_10_^+^ at *m*/*z* = 240, and all magnesium cluster ions larger than Mg_13_^+^ are the prevailing ions in the mass spectrum. The inset in the right corner of Figure 1 shows a section of this mass spectrum in the range of Mg_12_^+^. Between mass peaks close to integer mass per charge values that are predominantly formed by Mg_12_^+^ (blue circles), additional less intense mass peaks are found and can be assigned to Mg_24_^2+^ (red squares) and Mg_36_^3+^ (green triangles).

Utilizing the home-built software IsotopeFit,^67^ the yield of all Mg_*n*_^*z*+^ ions can be determined, including cluster sizes that are hidden between the intense He_*k*_^+^ cluster ions. The values obtained by IsotopeFit account for the isotopic pattern due to the natural abundance of the three stable magnesium isotopes, a background signal resulting from intense neighboring ion peaks, and potential isobaric ions due to impurities, such as complexes containing water picked up from the residual gas. The bottom diagram in Figure 1 shows the cluster size distribution of singly charged magnesium cluster ions, where the yields of Mg_*n*_^+^ are plotted as a function of the cluster size *n*. The data obtained by IsotopeFit from the above-mentioned mass spectrum (black solid circles) are compared with the cluster size distribution reported by Diederich et al. upon photoionization of magnesium-doped HNDs (average size 10^5^) with ns laser pulses at 266 nm^43^ (black open circles). Both cluster size distributions exhibit the same electronic shell closures and intensity anomalies in this mass range. Additionally, the cluster size distributions of doubly charged magnesium cluster ions are presented in the same figure. The red solid squares were retrieved by IsotopeFit from the mass spectrum shown in the upper diagram of Figure 1, and the red open squares were taken from the literature.^68^ The latter values were obtained from the isotopically resolved patterns of Mg_*n*_^2+^ ions that were formed via the ionization of Mg-doped HNDs with femtosecond laser pulses. Please note that the scale of the *x*-axis of the lower two diagrams is chosen to match the mass peaks in the upper diagram with the cluster size in the lower two diagrams.

#### 3.1.2 Mixed Magnesium C_60_ Cluster Ions

**Figure 2:**
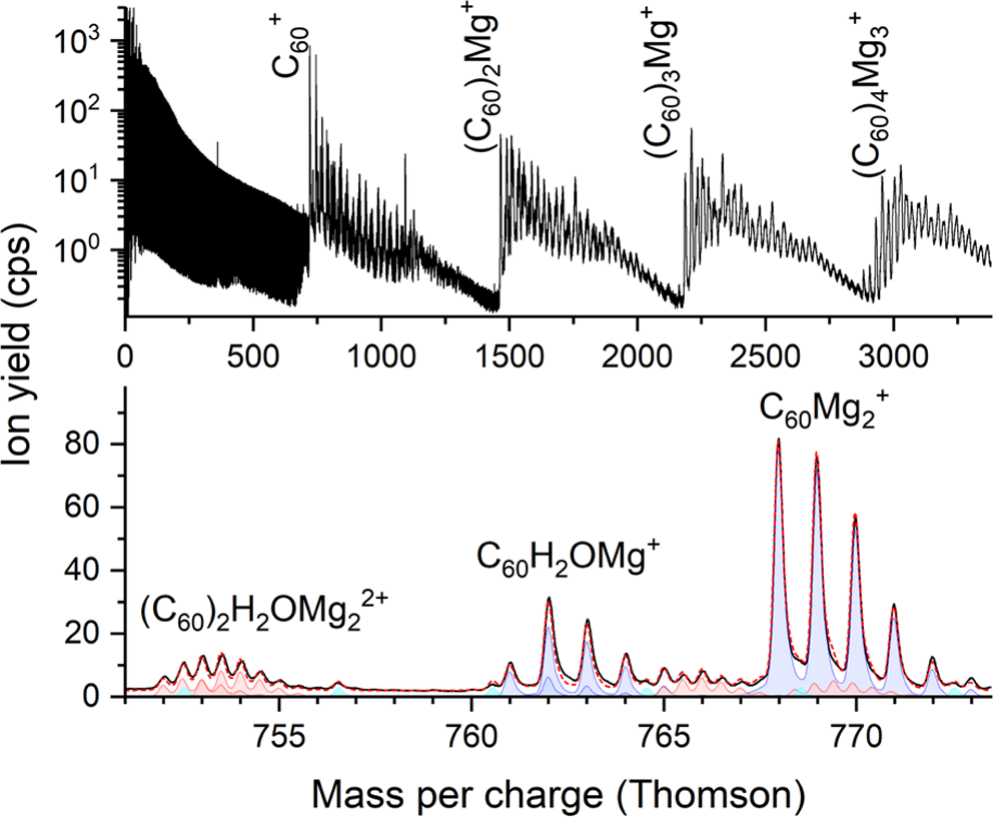
Mass spectrum of low-mass ions ejected from neutral HNDs doped with C_60_ and magnesium upon multiple electron bombardment (upper diagram). At mass per charge values below 720 Thomson, the mass spectrum is dominated by He_*k*_^+^ ions, exhibiting a biexponential decrease in ion yields as a function of *k*. At higher mass per charge values, the prevailing ions are (C_60_)_*m*_Mg_*n*_^+^. Pronounced intensity anomalies can be seen in this semilogarithmic plot. The lower diagram shows a section of the mass spectrum above (solid black line) and the resulting fit from IsotopeFit (red dashed line). The isotopic patterns of selected ions are included by taking the peak shape of the mass spectrometer into consideration. The most intense ions are indicated in the diagram. The detailed assignment of all ions can be found in Figure S1 of the Supporting Information (SI).

**Figure 3:**
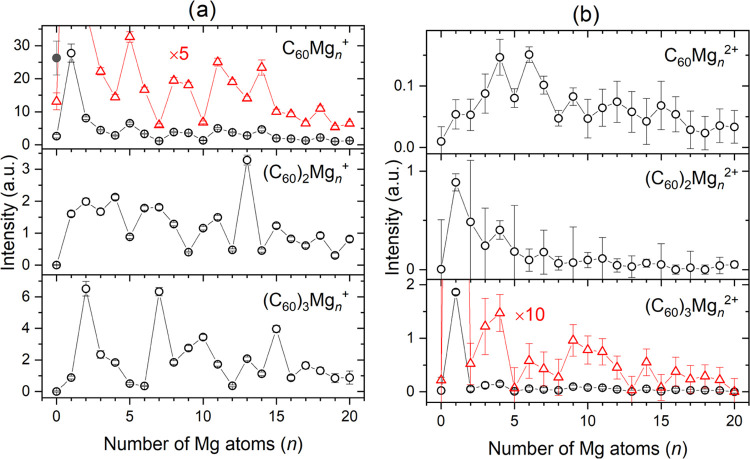
Cluster size distributions of (C_60_)_*m*_Mg_*n*_^+^ (a) and (C_60_)_*m*_Mg_*n*_^2+^ (b) as a function of the number of magnesium atoms (*n*) for *m* = 1–3. The ion yields were obtained by fitting the mass spectrum of Figure 2 with IsotopeFit^67^ taking into consideration the isotopic patterns and potential background signal, as well as the contributions from other ions in the mass range of individual mixed C_60_ magnesium cluster ions. The main contribution of C_60_^+^ (gray solid circle in the upper, left diagram) originates from the electron ionization of C_60_ molecules of an effusive fullerene beam emitted from the oven. Subtraction of these ions leads to a small contribution from doped HNDs that is prone to large error. To increase the visibility of the ion abundance of low-intensity cluster ions, the values were multiplied by a factor of 10 (open red triangles).

The mass spectrum shown in Figure 2 was measured under identical conditions as the one shown in Figure 1, but the fullerene oven was heated to 516 K. At this temperature, up to 10 fullerenes are picked up by the HNDs prior to doping with Mg. Again, the low-mass range is dominated by He_*k*_^+^ ions. At *m*/*z* = 360, C_60_^2+^ exceeds the pristine helium cluster ion series; however, this ion is formed upon electron ionization of gas phase C_60_ diffusing from the oven to the ion source. Also, C_60_^+^ most likely originates from electron ionization of C_60_ vapor, reaching the ion source. However, all cluster ions of the form (C_60_)_*m*_Mg_*n*_^+^ (*m* + *n* > 1) are fragment ions ejected from doped HNDs that are multiply charged by electron bombardment. The lower diagram of Figure 2 shows a section of the measured (black line) and fitted mass spectra (red dashed line) obtained by IsotopeFit. Three ions with the highest yield in the corresponding mass range were labeled. The shaded areas depict the contributions of different ions: singly charged ions in blue, doubly charged ions in red, and He_*k*_^+^ in cyan. The perfect agreement between the fitted and measured mass spectrum indicates that no additional ions have to be included. The cluster size distributions of singly and doubly charged (C_60_)_*m*_Mg_*n*_ ions for *m* = 1–3 as a function of *n* (*n* up to 20) obtained by IsotopeFit are plotted in Figure 3(a,b), respectively.

### 3.2 Ion Efficiency Curves

**Figure 4:**
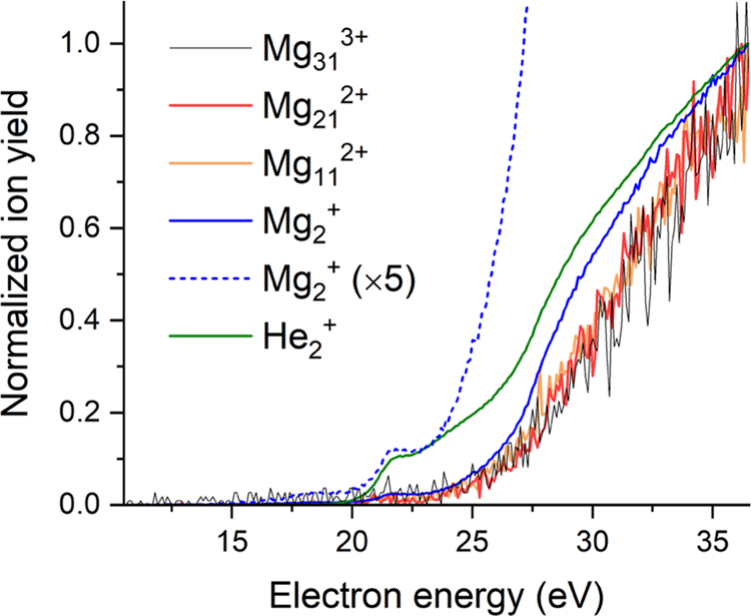
Normalized ion efficiency curves of selected monocations formed upon multiple electron bombardment of neutral HNDs doped with C_60_ and Mg. The singly charged dimer ions of He and Mg exhibit a pronounced shoulder at an electron energy of 22 eV that is missing for doubly and triply charged magnesium cluster ions. The ion efficiency curve of Mg_2_^+^ (blue solid line) was multiplied by a factor of 5 (dashed blue line) for better visibility of the 22 eV shoulder.

**Figure 5:**
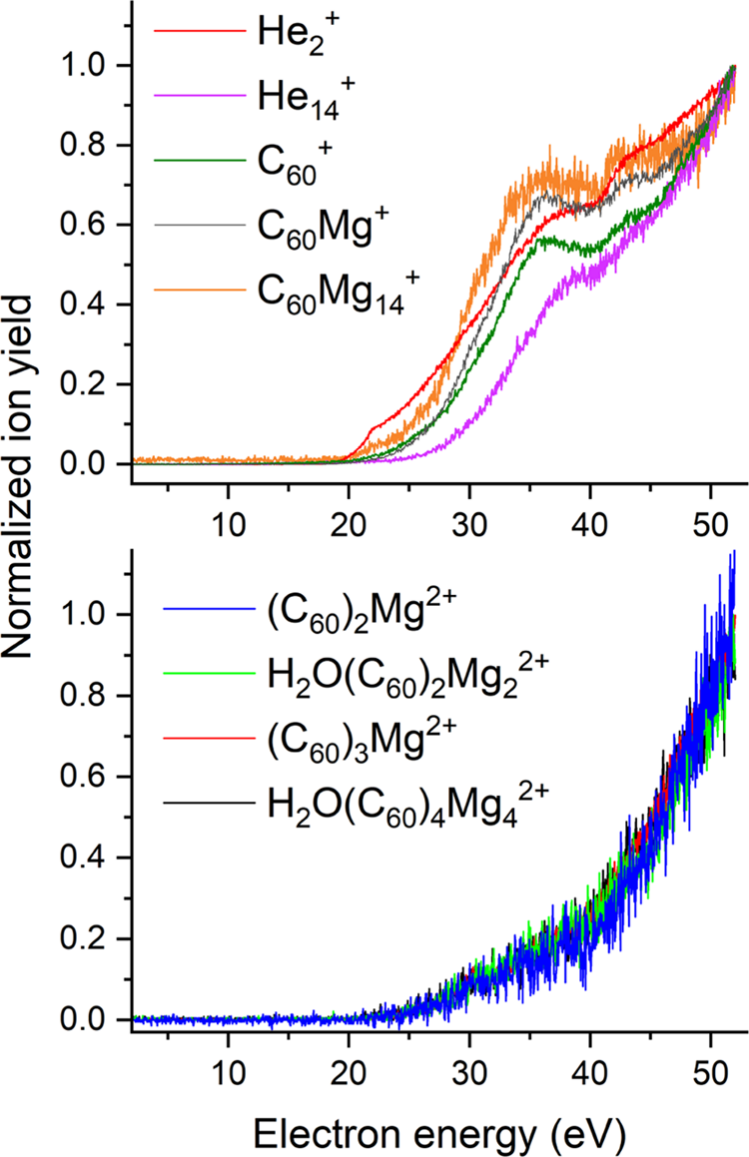
Ion efficiency curves for selected monocations (upper diagram) and dications (lower diagram) formed upon multiple electron bombardments of neutral HNDs doped with C_60_ and Mg. All singly charged ions except for He_14_^+^ exhibit a threshold at 20 eV that indicates an ionization mechanism that involves He*. In contrast, the dications are predominantly formed at electron energies higher than 24.6 eV, and all curves exhibit a pronounced signal rise at 40 eV, which indicates an additional ionization mechanism at electron energies higher than 40 eV.

In addition to mass spectra that were measured under constant parameters, we also measured all of the ion yields as a function of the electron energy. Ion efficiency curves for selected cations are shown in Figures 4 and 5.

#### 3.2.1 Pristine Magnesium Cluster Ions

The ion efficiency curves of Mg_2_^+^ (blue solid and dashed lines in Figure 4) and He_2_^+^ (green solid line in Figure 4) exhibit the same shoulder at an electron energy of 22 eV. At this electron energy, metastable He*^–^ is efficiently formed,^69^ and Renzler et al. observed the formation of He^+^ and He_2_^+^ at this energy via Penning ionization of He*^–^ by He* formed by a second electron.^70^ Also Penning ionization of neutral dopants by He* is energetically possible at this electron energy. The ion efficiency curves of doubly and triply charged magnesium cluster ions have a pronounced threshold energy of roughly 25 eV which is close to the ionization energy of helium at 24.6 eV. Note that the ion yields of the two dimer cations exhibit a clear increase at this energy, indicating the presence of charge transfer ionization via an initially formed He^+^ at electron energies above this value.

#### 3.2.2 Mixed Magnesium C_60_ Cluster Ions

In the upper diagram of Figure 5, normalized ion efficiency curves for selected monocations are plotted. He_2_^+^ again exhibits the shoulder at 22 eV discussed above, whereas all other cations are predominantly formed at electron energies higher than 24.6 eV. Furthermore, at an electron energy of about 40 eV, all curves display an increase in their slope, indicating the onset of a second ion formation channel.

The normalized ion efficiency curves of all dications (four selected ions are shown in the lower diagram of Figure 5) are basically identical. These ions are predominantly formed at electron energies higher than the ionization energy of He, and the signal rise above 40 eV is much more pronounced compared to monocations, indicating an additional ionization mechanism with a threshold energy of 40 eV.

## 4 Discussion

Pickup into HNDs is governed by the Poisson statistics, and in combination with a typical log-normal size distribution of neutral HNDs,^71−74^ the resulting cluster size distribution of a dopant X is expected to be log-normally distributed too. Furthermore, the binding energy released during the dopant cluster formation process is transferred to the surrounding helium matrix and dissipated via the evaporation of He atoms from the surface of the HNDs. As a result, the cluster size distributions of the dopants embedded in HNDs are smooth and free of local intensity anomalies. This was recently confirmed for the growth of charged cluster ions via pickup into previously charged HNDs.^75^ The pronounced intensity anomalies of the Mg_*n*_^*z*+^ and (C_60_)_*m*_Mg_*n*_^*z*+^ cluster series (shown in Figures 1 and 3) are the result of fragmentation during and after the ionization process, respectively. Charge transfer from He^+^ to a dopant cluster X_*N*_ (where *N* indicates the size of the neutral cluster) will transfer the difference of the ionization energies of He and the dopant cluster, as well as the difference of the solvation energies of He^+^ and the X_*N*_^+*^ surrounded by He atoms, into the internal degrees of freedom of the dopant cluster ion. This excess energy may lead to (i) the formation of doubly and triply charged cluster ions if it exceeds the respective ionization energies, (ii) the fragmentation of X_*N*_^+*^, or (iii) the evaporation of 1600 He atoms per eV energy that is transferred to the surrounding He matrix.

### 4.1 Pristine Magnesium Cluster Ions

So far, only very few doubly charged monomer ions X^2+^ have been observed upon ionization of doped neutral HNDs. In the case of atomic dopants, for a limited number of elements, the sum of the first and second ionization energies is low enough to enable dication formation via charge transfer from He^+^. The formation of highly charged atomic ions was observed upon strong field laser excitation of metal-doped HNDs utilizing either temporally stretched laser pulses or dual pulses.^76^ For electron ionization of doped HNDs, X^2+^ is additionally formed by double ionization of gas phase dopants X that reach the ion source. However, He_*k*_X^2+^ ions are unambiguously formed in HNDs. In the case of Ca, multiple ordered solvation shells were observed experimentally via intensity drops of the ion yield of He_*k*_Ca^2+^ at *k* = 12, 32, 44, and 74.^77^ In the mass spectrum shown in Figure 1, He_*k*_Mg^2+^ ions can be identified via the ^25^Mg isotopes up to *k* = 12; however, these ions are formed with low abundance. It is interesting to note that, so far, the formation of a molecular dication via ionization of neutral-doped HNDs has never been observed.

In contrast, the formation of doubly charged cluster ions of both polarities,^36,44,68,78−83^ as well as of triply charged cesium cluster ions,^82^ has been reported in the literature. The critical size, i.e., the smallest observable multiply charged cluster ions X_*n*_^*z*+^ formed in HNDs, is often substantially lower than for X_*n*_^*z*+^ formed upon ionization of pristine neutral clusters,^79,82^ indicating that the temperature of multiply charged cluster ions strongly affects their stability when approaching the Rayleigh limit. Döppner et al. reported the formation of Mg_*n*_^2+^ upon photoionization of neutral HNDs doped with magnesium and found Mg_5_^2+^ as the smallest dicationic cluster.^44^ In the present study, doubly and triply charged magnesium cluster ions are observed, with Mg_2_^2+^ and Mg_25_^3+^ being the smallest members, respectively. However, it has to be mentioned that the ion yields of doubly charged magnesium cluster ions with *n* < 5 are substantially smaller than those of larger cluster ions (see lower diagram of Figure 1). Saito et al. observed doubly charged magnesium dimer and trimer cluster ions from a liquid metal source, and they argue that Mg_2_^2+^ is expected to be covalently bound with a filled σ and an empty σ* orbital.^84^ Hogreve determined from multireference configuration interaction methods the structure and stability of Mg_2_^2+^ and found that the ground state of this dication supports many vibrational levels that are almost stable against dissociative tunneling.^85^

In the present experiment, the doped HNDs pass through the electron beam in about 5 μs. During this time an HND containg half a million He atoms is hit by roughly seven electrons at an electron energy of 50 eV, leading to the formation of four He^+^ cations and one He* based on the cross-section values for ionization and excitation taken from Bogdanov et al.^86^ Coulomb repulsion quickly locates cations in multiply charged HNDs close to the surface of the droplets and, furthermore, prevents the approach of He^+^ to a cationic dopant. In contrast, He* which is highly polarizable compared to ground state He and heliophobic, will be attracted to ions, and due to the surface location of the charge centers, it is expected to efficiently Penning ionize dopant cluster ions into higher charge states. The relatively long time span between individual ionization events where the ions have time to relax and even locate in low-energy configurations inside the HNDs differs strongly from the processes driven by nanosecond and femtosecond laser pulses.^43^ Nevertheless, the remarkable similarity between mass spectra obtained upon photoionization and electron ionization of neutral HNDs doped with Mg and the pronounced intensity anomalies indicate that both ionization methods cause fragmentation due to excess energy transferred to the interatomic vibrations of initially doubly charged magnesium cluster ions. Cluster ions with electronic shell closures, as known from alkali metal clusters, are more stable than their neighbors and thus are observed as local intensity maxima in the cluster size distributions (see Figure 1b,c). Applying a level interchange model based on the local density approximation, Diederich et al. were able to obtain a reasonable explanation for the measured cluster size distribution.^43^ Pristine magnesium clusters, both singly and doubly charged, exhibit magic numbers in the cluster size distributions that suggest full electron delocalization of the two valence electrons of each Mg atom at a cluster size of about *n* = 20.^43^ This is also the cluster size at which the nonmetal-to-metal transition is expected.^87,88^

### 4.2 Mixed Magnesium C_60_ Cluster Ions

The cluster size distributions of (C_60_)_*m*_Mg_*n*_^*z*+^ ions (*z* = 1 and 2) as a function of the number of magnesium atoms *n* for one to three fullerenes exhibit some pronounced local intensity maxima that depend on the charge state and the number of fullerenes (*m*). Table 1 summarizes all particularly intense (C_60_)_*m*_Mg_*n*_^*z*+^ (singly charged ions are before and doubly charged ions after a slash), and the most pronounced intensity anomalies are underlined.

**Table 1:** Number of Magnesium Atoms *n* of (C_60_)_*m*_Mg_*n*_^+^/(C_60_)_*m*_Mg_*n*_^2+^ that Exhibit Local Intensity Anomalies (Magic Numbers)^a^

*m* =	0	1	2	3	4	5	6	7
	5/11	1/4	2/1	2/1	3	8	10	15
	10/16	5/6	4/4	7/6	6	18		
	20/18	8/9	6	10/9	10			
	25/21	11/12	13	15/14	12			
	30/30	14/15	18	21	14			
	35/36	32	23		24			

a Underlined numbers indicate particularly intense cluster ions.

In a second mass spectrum measured at a higher temperature of the magnesium oven (shown in the Figure S2 of the SI), decoration of C_60_ with more than 32 Mg atoms could be achieved to investigate for a magic number at a commensurate coverage of the fullerene cage, similar to what was observed in the case of the heavy alkaline earth metals Ca, Sr, and Ba.^27^ C_60_Mg_32_^+^ is indeed almost twice as intense as C_60_Mg_*n*_^+^ with *n* > 32. This indicates that magnesium is wetting the fullerene surface due to the fact that the binding energy between C_60_ and Mg is higher than the binding energy between two Mg atoms, which was measured to be only 50 meV.^89^ According to Robledo et al., the charge of a C_60_Mg^+^ complex is almost completely located at the metal atom, and the binding energy of Mg^+^ to C_60_ is 1.37 eV.^21^ This is comparable to the binding energy of a magnesium atom in bulk with values between 1.4^90^ and 1.51 eV^91^, which can be found in the literature. In order to obtain a commensurate 1 × 1 decoration of the 32 C_60_ faces, a transition from the first Mg^+^ ion from a position above an edge formed by a pentagonal and hexagonal face^21^ to a position above the center of a pentagonal or hexagonal face.

In the case of (C_60_)_*m*_Na_*n*_^+/2+^, six sodium atoms per C_60_ and one or two additional sodium ions for the charge of singly and doubly charged cluster, respectively, are particularly abundant ions followed by an odd–even oscillation well-known in size distributions of pristine alkali clusters.^33,37^ In contrast, no corresponding patterns can be found for the (C_60_)_*m*_Mg_*n*_^+/2+^ complexes.

Zöttl et al. discovered that atomic and molecular dopants that are weakly interacting with C_60_ preferentially adsorb at dimple sites formed by three carbon cages followed by positions at grooves formed by two fullerenes.^31,92^ The particular magic number at *n* = 2 for *m* = 3 agrees with this observation. The tetramer of C_60_ is expected to have a tetrahedral structure, with each of the four faces representing a dimple site. The most prominent magic number, however, is found at *n* = 6. Clusters containing *m* = 5 and 6 fullerenes are most likely formed in bipyramidal structures with 6 and 8 faces, respectively, and each of them representing a dimple site. However, the magic numbers observed in the experiment are 8 and 10, respectively. A possible tentative explanation for these magic numbers is a positively charged magnesium dimer in the voids formed by the fullerene cages and one Mg atom occupying each dimple site at the surface of the pyramidal structures. However, only sophisticated quantum chemical calculations can give a definite answer about the structure of these magic (C_60_)_*m*_Mg_*n*_^+^ clusters.

### 4.3 Ion Efficiency Curves and Ionization Mechanisms

Magnesium and C_60_ both tend to form clusters within HNDs, which typically have an average size of about 500,000 helium atoms. However, despite this usual clustering behavior, there is evidence showing that a small number of magnesium atoms can create a foam within these HNDs.^48,56^

The size of typical HNDs utilized in the present experiments is quite large, and for a spherical shape, the geometric cross-section is 1640 nm^2^. From the velocity of these droplets, which was determined to be 190 m/s,^71^ the electron current (132 μA), and the diameter of the electron current (1 mm), we can determine that these droplets are hit by about 7 electrons. At sufficiently high electron energies, this may lead to the formation of several He^+^ and He*, and at some specific electron energies also to He*^–^.^69^ All three of these species have enough energy to ionize a (C_60_)_*M*_Mg_*N*_ dopant cluster by either charge transfer or Penning ionization. Positively charged He^+^ ions will be repelled from the cationic dopant cluster, but He* and He*^–^ will be attracted by the ion-induced dipole and Coulomb interaction, respectively. The potential energy of these species is high enough to remove additional electrons from dopant clusters by Penning ionization, and the threshold of 40 eV matches the onset of sequential Penning ionization discovered by Schöbel et al. for atomic iodine.^66^ The ion efficiency curves of the singly charged dimers Mg_2_^+^ and He_2_^+^ (Figure 4) exhibit a shoulder at about 22 eV indicating the formation of an intermediate He*^–^ which is efficiently formed at this energy.^69^ At about 30 eV, the slope of the ion efficiency curves of both singly charged ions decreases, which is not observed for the doubly and triply charged magnesium cluster ions shown in Figure 4. This suggests an additional ionization mechanism for multiply charged ions with a threshold at about 30 eV that compensated for the reduction of the slope observed for singly charged ions. In the case of mixed C_60_ magnesium cluster ions, a similar trend is observed. All doubly charged ions (lower diagram of Figure 5) exhibit a much stronger increase in ion yield at electron energies larger than 40 eV compared to the singly charged ions shown in the upper diagram. This indicates that sequential Penning ionization is driving the formation of dications. Splitting of doubly charged cluster ions below a critical size into singly charged fragment ions is often called Coulomb explosion and can explain the less pronounced increase in the slope of the ion efficiency curves of small singly charged cluster ions above the threshold energy of sequential Penning ionization.

## 5 Conclusions

Pristine magnesium and mixed fullerene–magnesium cluster ions are formed upon the pickup of individual atoms and molecules into neutral HNDs that are subsequently exposed to multiple electron bombardments. Charge transfer from initially formed He_*n*_^+^ ionic cores, along with Penning ionization from metastable helium atoms, leads to multiply charged HNDs. This then results in the ejection of low-mass ions such as pristine helium cluster cations containing up to a few hundred He atoms, as well as singly to triply charged dopant cluster ions. The smallest doubly and triply charged magnesium cluster ions observed are Mg_2_^2+^ and Mg_25_^3+^, respectively.

Ion efficiency curves reveal details about the underlying ionization mechanisms. For singly charged magnesium cluster ions, an energy threshold appears at around 20 eV, indicating that Penning ionization is operational at this low electron energy. A pronounced increase in the ion yield at around 25 eV can be assigned to charge transfer from a He_*n*_^+^ charge center, which forms upon electron ionization of a single He atom. Multiply charged dopant cluster ions are exclusively formed via this second mechanism. In the case of fullerene–magnesium complexes, a pronounced kink in the ion efficiency curves at 40 eV suggests a sequential ionization mechanism. This involves a metastable He atom being attracted to a singly charged dopant cluster ion, with energy transfer leading to Penning ionization and the creation of a doubly charged species.^66,91^

Intensity anomalies in cluster size distributions of singly and doubly charged ions are obtained from high-resolution mass spectra utilizing sophisticated software that considers the complex isotopic patterns.^65^ The cluster size distributions of pristine singly and doubly charged magnesium cluster ions agree perfectly with mass spectra reported in the literature,^43^ exhibiting the same intensity anomalies due to electronic shell closures. In mixed fullerene–magnesium clusters, (C_60_)_3_Mg_2_^+^ and (C_60_)_3_Mg^2+^ turned out to be particularly intense ions. We tentatively assign a structure to the monocation where the two magnesium atoms are located at the two dimple sites formed by the three buckyballs, and in the case of the dication, we suggest that the reduced size of the Mg^2+^ ion fits right between the three buckyballs. However, only sophisticated quantum chemical calculations can give a definite answer, which is beyond the scope of this paper.

## Supporting Information Available

Figure S1: Assignment of all ions contributing to the mass spectrum section featured in the lower panel of Figure 2. Figure S2: Mass spectrum and cluster size distribution of C_60_Mg_*n*_^+^, with *n* up to 40 (PDF)

## References

[ref1] German E.; Hou G. L.; Vanbuel J.; Bakker J. M.; Alonso J. A.; Janssens E.; Lopez M. J. Infrared spectra and structures of C_60_Rh_n_^+^ complexes. Carbon 2022, 197, 535–543. 10.1016/j.carbon.2022.07.002.

[ref2] Suggs K.; Msezane A. Z. Doubly-Charged Negative Ions as Novel Tunable Catalysts: Graphene and Fullerene Molecules Versus Atomic Metals. Int. J. Mol. Sci. 2020, 21, 6714 10.3390/ijms21186714. 32933219 PMC7554846

[ref3] Afreen S.; Muthoosamy K.; Manickam S.; Hashim U. Functionalized fullerene C_60_ as a potential nanomediator in the fabrication of highly sensitive biosensors. Biosens. Bioelectron. 2015, 63, 354–364. 10.1016/j.bios.2014.07.044. 25125029

[ref4] Bashiri S.; Vessally E.; Bekhradnia A.; Hosseinian A.; Edjlali L. Utility of extrinsic [60] fullerenes as work function type sensors for amphetamine drug detection: DFT studies. Vacuum 2017, 136, 156–162. 10.1016/j.vacuum.2016.12.003.

[ref5] Zhao Y. F.; Kim Y. H.; Dillon A. C.; Heben M. J.; Zhang S. B. Hydrogen storage in novel organometallic buckyballs. Phys. Rev. Lett. 2005, 94, 155504 10.1103/PhysRevLett.94.155504. 15904160

[ref6] Kothandam G.; Singh G.; Guan X. W.; Lee J. M.; Ramadass K.; Joseph S.; Benzigar M.; Karakoti A.; Yi J. B.; Kumar P.; et al. Recent Advances in Carbon-Based Electrodes for Energy Storage and Conversion. Adv. Sci. 2023, 10, 2301045 10.1002/advs.202301045. PMC10288283 37096838

[ref7] Yoon M.; Yang S. Y.; Hicke C.; Wang E.; Geohegan D.; Zhang Z. Y. Calcium as the superior coating metal in functionalization of carbon fullerenes for high-capacity hydrogen storage. Phys. Rev. Lett. 2008, 100, 206806 10.1103/PhysRevLett.100.206806. 18518569

[ref8] Yue C. M.; Nomura Y.; Werner P. Doping Asymmetry and Layer-Selective Metal-Insulator Transition in Trilayer K_3+x_C_60_. Phys. Rev. Lett. 2022, 129, 066403 10.1103/PhysRevLett.129.066403. 36018629

[ref9] Hebard A. F.; Rosseinsky M. J.; Haddon R. C.; Murphy D. W.; Glarum S. H.; Palstra T. T. M.; Ramirez A. P.; Kortan A. R. Superconductivity at 18 K in potassium-doped C_60_. Nature 1991, 350, 600–601. 10.1038/350600a0. 10043627

[ref10] Lavrentiev V.; Chvostova D.; Pokorny J.; Lavrentieva I.; Vacik J.; Dejneka A. Tuneable interplay of plasmonic and molecular excitations in self-assembled silver-fullerene nanocomposites. Carbon 2021, 184, 34–42. 10.1016/j.carbon.2021.08.002.

[ref11] Guo J.; Si M. M.; Zhao X. T.; Wang L.; Wang K.; Hao J. Y.; Wang H.; Randall C. A. Altering interfacial properties through the integration of C_60_ into ZnO ceramic via cold sintering process. Carbon 2022, 190, 255–261. 10.1016/j.carbon.2022.01.017.

[ref12] Sun Q.; Jena P.; Wang Q.; Marquez M. First-principles study of hydrogen storage on Li_12_C_60_. J. Am. Chem. Soc. 2006, 128, 9741–9745. 10.1021/ja058330c. 16866529

[ref13] Li Y. F. Molecular Design of Photovoltaic Materials for Polymer Solar Cells: Toward Suitable Electronic Energy Levels and Broad Absorption. Acc. Chem. Res. 2012, 45, 723–733. 10.1021/ar2002446. 22288572

[ref14] Shinohara H. Endohedral metallofullerenes. Rep. Prog. Phys. 2000, 63, 843–892. 10.1088/0034-4885/63/6/201.

[ref15] Yang S. F.; Wei T.; Jin F. When metal clusters meet carbon cages: endohedral clusterfullerenes. Chem. Soc. Rev. 2017, 46, 5005–5058. 10.1039/C6CS00498A. 28681052

[ref16] Heath J. R.; Obrien S. C.; Zhang Q.; Liu Y.; Curl R. F.; Kroto H. W.; Tittel F. K.; Smalley R. E. Lanthanum Complexes of Spheroidal Carbon Shells. J. Am. Chem. Soc. 1985, 107, 7779–7780. 10.1021/ja00311a102.

[ref17] Kroto H. W.; Heath J. R.; Obrien S. C.; Curl R. F.; Smalley R. E. C_60_ - Buckminsterfullerene. Nature 1985, 318, 162–163. 10.1038/318162a0.

[ref18] Chai Y.; Guo T.; Jin C. M.; Haufler R. E.; Chibante L. P. F.; Fure J.; Wang L. H.; Alford J. M.; Smalley R. E. Fullerenes with metals inside. J. Phys. Chem. A 1991, 95, 7564–7568. 10.1021/j100173a002.

[ref19] Weaver J. H.; Chai Y.; Kroll G. H.; Jin C.; Ohno T. R.; Haufler R. E.; Guo T.; Alford J. M.; Conceicao J.; Chibante L. P. F.; et al. XPS probes of carbon-caged metals. Chem. Phys. Lett. 1992, 190, 460–464. 10.1016/0009-2614(92)85173-8.

[ref20] Cioslowski J.; Fleischmann E. D. Endohedral complexes - atoms and ions inside the C_60_ cage. J. Chem. Phys. 1991, 94, 3730–3734. 10.1063/1.459744.

[ref21] Robledo M.; Aguirre N. F.; Diaz-Tendero S.; Martin F.; Alcami M. Bonding in exohedral metal-fullerene cationic complexes. RSC Adv. 2014, 4, 53010–53020. 10.1039/C4RA10776D.

[ref22] Stephens P. W.; Mihaly L.; Lee P. L.; Whetten R. L.; Huang S. M.; Kaner R.; Deiderich F.; Holczer K. Structure of single-phase superconducting K_3_C_60_. Nature 1991, 351, 632–634. 10.1038/351632a0.

[ref23] Zhou O.; Fischer J. E.; Coustel N.; Kycia S.; Zhu Q.; Mcghie A. R.; Romanow W. J.; Mccauley J. P.; Smith A. B.; Cox D. E. Structure and bonding in alkali-metal-doped C_60_. Nature 1991, 351, 462–464. 10.1038/351462a0.

[ref24] Zimmerman P. A.; Hercules D. M. Formation and Detection of Fullerene Metal Complexes Using Time-Of-Flight Secondary Ion Mass Spectrometry. Appl. Spectrosc. 1993, 47, 1545–1547. 10.1366/0003702934334651.

[ref25] Zimmermann U.; Malinowski N.; Burkhardt A.; Martin T. P. Metal-coated fullerenes. Carbon 1995, 33, 995–1006. 10.1016/0008-6223(95)00028-C.

[ref26] Springborg M.; Satpathy S.; Malinowski N.; Zimmermann U.; Martin T. P. Electronic shell structure and relative abundances of cesium-coated C_60_. Phys. Rev. Lett. 1996, 77, 1127–1130. 10.1103/PhysRevLett.77.1127. 10062997

[ref27] Zimmermann U.; Malinowski N.; Näher U.; Frank S.; Martin T. P. Multilayer metal coverage of fullerene molecules. Phys. Rev. Lett. 1994, 72, 3542–3545. 10.1103/PhysRevLett.72.3542. 10056226

[ref28] Kurikawa T.; Nagao S.; Miyajima K.; Nakajima A.; Kaya K. Formation of Cobalt-C_60_ Clusters: Tricapped Co(C_60_)_3_ Unit. J. Phys. Chem. A 1998, 102, 1743–1747. 10.1021/jp980209n.

[ref29] Leidlmair C.; Wang Y.; Bartl P.; Schöbel H.; Denifl S.; Probst M.; Alcami M.; Martin F.; Zettergren H.; Hansen K.; et al. Structures, Energetics, and Dynamics of Helium Adsorbed on Isolated Fullerene Ions. Phys. Rev. Lett. 2012, 108, 076101 10.1103/PhysRevLett.108.076101. 22401228

[ref30] Leidlmair C.; Bartl P.; Schöbel H.; Denifl S.; Probst M.; Scheier P.; Echt O. On the Possible Presence of Weakly Bound Fullerene-H_2_ Complexes in the Interstellar Medium. Astrophys. J., Lett. 2011, 738, L4 10.1088/2041-8205/738/1/L4.

[ref31] Zöttl S.; Kaiser A.; Bartl P.; Leidlmair C.; Mauracher A.; Probst M.; Denifl S.; Echt O.; Scheier P. Methane Adsorption on Graphitic Nanostructures: Every Molecule Counts. J. Phys. Chem. Lett. 2012, 3, 2598–2603. 10.1021/jz301106x. 23378887 PMC3560424

[ref32] Calvo F. Size-induced melting and reentrant freezing in fullerene-doped helium clusters. Phys. Rev. B 2012, 85, 060502 10.1103/PhysRevB.85.060502.

[ref33] Martin T. P.; Malinowski N.; Zimmermann U.; Näher U.; Schaber H. Metal-coated fullerene molecules and clusters. J. Chem. Phys. 1993, 99, 4210–4212. 10.1063/1.466118.

[ref34] Hou G. L.; Lushchikova O. V.; Bakker J. M.; Lievens P.; Decin L.; Janssens E. Buckyball-metal Complexes as Potential Carriers of Astronomical Unidentified Infrared Emission Bands. Astrophys. J. 2023, 952, 13 10.3847/1538-4357/accf1b.

[ref35] Xu J.; Bakker J. M.; Lushchikova O. V.; Lievens P.; Janssens E.; Hou G. L. Pentagon, Hexagon, or Bridge? Identifying the Location of a Single Vanadium Cation on Buckminsterfullerene Surface. J. Am. Chem. Soc. 2023, 145, 22243–22251. 10.1021/jacs.3c08451. 37757468

[ref36] Mauracher A.; Echt O.; Ellis A. M.; Yang S.; Bohme D. K.; Postler J.; Kaiser A.; Denifl S.; Scheier P. Cold Physics and Chemistry: Collisions, Ionization and Reactions inside Helium Nanodroplets Close to Zero K. Phys. Rep. 2018, 751, 1–90. 10.1016/j.physrep.2018.05.001.

[ref37] Harnisch M.; Daxner M.; Scheier P.; Echt O. Adsorption of sodium and cesium on aggregates of C_60_. Eur. Phys. J. D 2016, 70, 192 10.1140/epjd/e2016-70438-4.

[ref38] Renzler M.; Kranabetter L.; Goulart M.; Scheier P.; Echt O. Positively and Negatively Charged Cesium and (C_60_)_m_Cs_n_ Cluster Ions. J. Phys. Chem. C 2017, 121, 10817–10823. 10.1021/acs.jpcc.6b11928. PMC5447244 28572870

[ref39] Kaiser A.; Renzler M.; Kranabetter L.; Schwarzler M.; Parajuli R.; Echt O.; Scheier P. On enhanced hydrogen adsorption on alkali (cesium) doped C_60_ and effects of the quantum nature of the H_2_ molecule on physisorption energies. Int. J. Hydrogen Energy 2017, 42, 3078–3086. 10.1016/j.ijhydene.2017.01.069.

[ref40] Goulart M.; Kuhn M.; Martini P.; Chen L.; Hagelberg F.; Kaiser A.; Scheier P.; Ellis A. M. Highly Stable C_60_AuC_60_^±^ Dumbbells. J. Phys. Chem. Lett. 2018, 9, 2703–2706. 10.1021/acs.jpclett.8b01047. 29722981 PMC5964450

[ref41] Martini P.; Goulart M.; Kranabetter L.; Gitzl N.; Rasul B.; Scheier P.; Echt O. Charged Clusters of C_60_ and Au or Cu: Evidence for Stable Sizes and Specific Dissociation Channels. J. Phys. Chem. A 2019, 123, 4599–4608. 10.1021/acs.jpca.9b02768. 31062979 PMC6545602

[ref42] Nauta K.; Moore D. T.; Stiles P. L.; Miller R. E. Probing the structure of metal cluster-adsorbate systems with high-resolution infrared spectroscopy. Science 2001, 292, 481–484. 10.1126/science.1058896. 11313489

[ref43] Diederich T.; Döppner T.; Braune J.; Tiggesbäumker J.; Meiwes-Broer K. H. Electron delocalization in magnesium clusters grown in supercold helium droplets. Phys. Rev. Lett. 2001, 86, 4807–4810. 10.1103/PhysRevLett.86.4807. 11384353

[ref44] Döppner T.; Diederich T.; Tiggesbäumker J.; Meiwes-Broer K. H. Femtosecond ionization of magnesium clusters grown in ultracold helium droplets. Eur. Phys. J. D 2001, 16, 13–16. 10.1007/s100530170049. 11384353

[ref45] Stiles P. L.; Moore D. T.; Miller R. E. Structures of HCN-Mg_n_ (n = 2–6) complexes from rotationally resolved vibrational spectroscopy and ab initio theory. J. Chem. Phys. 2004, 121, 3130–3142. 10.1063/1.1768932. 15291623

[ref46] Przystawik A.; Göde S.; Döppner T.; Tiggesbäumker J.; Meiwes-Broer K. H. Light-induced collapse of metastable magnesium complexes formed in helium nanodroplets. Phys. Rev. A 2008, 78, 021202(R) 10.1103/PhysRevA.78.021202.

[ref47] Krasnokutski S. A.; Huisken F. Ultra-Low-Temperature Reactions of Mg Atoms with O_2_ Molecules in Helium Droplets. J. Phys. Chem. A 2010, 114, 7292–7300. 10.1021/jp103947z. 20560588

[ref48] Göde S.; Irsig R.; Tiggesbäumker J.; Meiwes-Broer K. H. Time-resolved studies on the collapse of magnesium atom foam in helium nanodroplets. New J. Phys. 2013, 15, 015026 10.1088/1367-2630/15/1/015026.

[ref49] Emery S. B.; Rider K. B.; Little B. K.; Lindsay C. M. Helium Droplet Assembled Nanocluster Films: Cluster Formation and Deposition Rates. J. Phys. Chem. C 2013, 117, 2358–2368. 10.1021/jp310295h.

[ref50] Emery S. B.; Rider K. B.; Little B. K.; Schrand A. M.; Lindsay C. M. Magnesium cluster film synthesis by helium nanodroplets. J. Chem. Phys. 2013, 139, 054307 10.1063/1.4817326. 23927262

[ref51] Emery S. B.; Rider K. B.; Lindsay C. M. Stabilized Magnesium/ Perfluoropolyether Nanocomposite Films by Helium Droplet Cluster Assembly. Propellants, Explos. Pyrotech. 2014, 39, 161–165. 10.1002/prep.201300158.

[ref52] Emery S. B.; Xin Y.; Ridge C. J.; Buszek R. J.; Boatz J. A.; Boyle J. M.; Little B. K.; Lindsay C. M. Unusual behavior in magnesium-copper cluster matter produced by helium droplet mediated deposition. J. Chem. Phys. 2015, 142, 084307 10.1063/1.4913210. 25725731

[ref53] Laforge A. C.; Stumpf V.; Gokhberg K.; Von Vangerow J.; Stienkemeier F.; Kryzhevoi N. V.; O’keeffe P.; Ciavardini A.; Krishnan S. R.; Coreno M.; et al. Enhanced Ionization of Embedded Clusters by Electron-Transfer-Mediated Decay in Helium Nanodroplets. Phys. Rev. Lett. 2016, 116, 203001 10.1103/PhysRevLett.116.203001. 27258866

[ref54] Kazak L.; Göde S.; Meiwes-Broer K. H.; Tiggesbäumker J. Photoelectron Spectroscopy on Magnesium Ensembles in Helium Nanodroplets. J. Phys. Chem. A 2019, 123, 5951–5956. 10.1021/acs.jpca.9b02880. 31240915

[ref55] Krebs B. S.; Tulsky V.; Kazak L.; Zabel M.; Bauer D.; Tiggesbäumker J. Phase-of-the-Phase Electron Momentum Spectroscopy on Single Metal Atoms in Helium Nanodroplets. J. Phys. Chem. Lett. 2022, 13, 1526–1532. 10.1021/acs.jpclett.2c00110. 35133167

[ref56] Kazak L.; Meiwes-Broer K. H.; Tiggesbäumker J. Ionization potentials of Mg_N_ (N = 7–56) clusters formed by spontaneous collapse of magnesium foam in helium nanodroplets. Phys. Chem. Chem. Phys. 2022, 24, 23350–23356. 10.1039/D2CP03075F. 36134466

[ref57] Hernando A.; Barranco M.; Mayol R.; Pi M.; Ancilotto F. Density functional theory of the structure of magnesium-doped helium nanodroplets. Phys. Rev. B 2008, 78, 184515 10.1103/PhysRevB.78.184515.

[ref58] Snyder D. N.; Szczesniak M. M.; Chalasinski G. The nature of interactions between clusters of Mg and Zn with HCN from symmetry-adapted perturbation theory based of DFT. J. Chem. Phys. 2009, 130, 224704 10.1063/1.3152122. 19530781

[ref59] Navarro J.; Mateo D.; Barranco M.; Sarsa A. Mg impurity in helium droplets. J. Chem. Phys. 2012, 136, 054301 10.1063/1.3675919. 22320736

[ref60] Höller J.; Krotscheck E.; Zillich R. E. Mg and Na clusters in a helium matrix. Eur. Phys. J. D 2015, 69, 198 10.1140/epjd/e2015-60280-7.

[ref61] Buszek R. J.; Ridge C. J.; Emery S. B.; Lindsay C. M.; Boatz J. A. Theoretical Study of Cu/Mg Core-shell Nanocluster Formation. J. Phys. Chem. A 2016, 120, 9612–9617. 10.1021/acs.jpca.6b09772. 27933919

[ref62] Mohajeri A. Mg/Cu bimetallic nanoalloys: Morphologies, electronic structures, and catalysis of O_2_ dissociation. J. Alloys Compd. 2018, 735, 1962–1970. 10.1016/j.jallcom.2017.11.328.

[ref63] Emery S. B.; Boyle J. M.; Rider K. B.; Little B. K.; Lindsay C. M. Nano-scale energetic films by superfluid helium droplet assembly. J. Phys.: Conf. Ser. 2014, 500, 052012 10.1088/1742-6596/500/5/052012.

[ref64] Stiles P. L.; Miller R. E. Structures and bonding nature of small monoligated copper clusters (HCN-Cu_n_, n = 1–3) through high-resolution infrared spectroscopy and theory. J. Phys. Chem. A 2006, 110, 10225–10235. 10.1021/jp063187a. 16928112

[ref65] Schöbel H.; Bartl P.; Leidlmair C.; Denifl S.; Echt O.; Märk T. D.; Scheier P. High-resolution mass spectrometric study of pure helium droplets, and droplets doped with krypton. Eur. Phys. J. D 2011, 63, 209–214. 10.1140/epjd/e2011-10619-1.

[ref66] Schöbel H.; Bartl P.; Leidlmair C.; Daxner M.; Zöttl S.; Denifl S.; Märk T. D.; Scheier P.; Spångberg D.; Mauracher A.; et al. Sequential Penning Ionization: Harvesting Energy with Ions. Phys. Rev. Lett. 2010, 105, 243402 10.1103/PhysRevLett.105.243402. 21231525

[ref67] Ralser S.; Postler J.; Harnisch M.; Ellis A. M.; Scheier P. Extracting cluster distributions from mass spectra: IsotopeFit. Int. J. Mass Spectrom. 2015, 379, 194–199. 10.1016/j.ijms.2015.01.004. 26109907 PMC4461193

[ref68] Diederich T.; Döppner T.; Fennel T.; Tiggesbäumker J.; Meiwes-Broer K. H. Shell structure of magnesium and other divalent metal clusters. Phys. Rev. A 2005, 72, 023203 10.1103/PhysRevA.72.023203.

[ref69] Mauracher A.; Daxner M.; Postler J.; Huber S. E.; Denifl S.; Scheier P.; Toennies J. P. Detection of Negative Charge Carriers in Superfluid Helium Droplets: The Metastable Anions He*^–^ and He_2_*^–^. J. Phys. Chem. Lett. 2014, 5, 2444–2449. 10.1021/jz500917z. 25068008 PMC4106244

[ref70] Renzler M.; Daxner M.; Weinberger N.; Denifl S.; Scheier P.; Echt O. On subthreshold ionization of helium droplets, ejection of He^+^, and the role of anions. Phys. Chem. Chem. Phys. 2014, 16, 22466–22470. 10.1039/C4CP03236E. 25230760

[ref71] Laimer F.; Zappa F.; Scheier P. Size and Velocity Distribution of Negatively Charged Helium Nanodroplets. J. Phys. Chem. A 2021, 125, 7662–7669. 10.1021/acs.jpca.1c05619. 34449223 PMC9282675

[ref72] Gomez L. F.; Loginov E.; Sliter R.; Vilesov A. F. Sizes of large He droplets. J. Chem. Phys. 2011, 135, 154201 10.1063/1.3650235. 22029306

[ref73] Sliter R.; Gomez L. F.; Kwok J.; Vilesov A. Sizes distributions of large He droplets. Chem. Phys. Lett. 2014, 600, 29–33. 10.1016/j.cplett.2014.03.053.

[ref74] Toennies J. P.; Vilesov A. F. Superfluid helium droplets: a uniquely cold nanomatrix for molecules and molecular complexes. Angew. Chem., Int. Ed. 2004, 43, 2622–2648. 10.1002/anie.200300611. 18629978

[ref75] Kollotzek S.; Lushchikova O. V.; Tiefenthaler L.; Zappa F.; Scheier P. Efficient Formation of Size-Selected Clusters upon Pickup of Dopants into Multiply Charged Helium Droplets. Int. J. Mol. Sci. 2022, 23, 3613 10.3390/ijms23073613. 35408968 PMC8998201

[ref76] Döppner T.; Diederich T.; Przystawik A.; Truong N. X.; Fennel T.; Tiggesbäumker J.; Meiwes-Broer K. H. Charging of metal clusters in helium droplets exposed to intense femtosecond laser pulses. Phys. Chem. Chem. Phys. 2007, 9, 4639–4652. 10.1039/b703707d. 17700865

[ref77] Zunzunegui-Bru E.; Gruber E.; Lázaro T.; Bartolomei M.; Hernández M. I.; Campos-Martínez J.; González-Lezana T.; Bergmeister S.; Zappa F.; Scheier P.; et al. Observation of Multiple Ordered Solvation Shells in Doped Helium Droplets. J. Phys. Chem. Lett. 2023, 14, 3126–3131. 10.1021/acs.jpclett.3c00224. 36952614 PMC10084467

[ref78] Martini P.; Kranabetter L.; Goulart M.; Kuhn M.; Gatchell M.; Bohme D. K.; Scheier P. Formation of positive and negative clusters of gold atoms inside helium nanodroplets close to zero K. Int. J. Mass Spectrom. 2018, 434, 136–141. 10.1016/j.ijms.2018.09.020.

[ref79] Mahmoodi-Darian M.; Raggl S.; Renzler M.; Goulart M.; Huber S. E.; Mauracher A.; Scheier P.; Echt O. Doubly charged coronene clusters-Much smaller than previously observed. J. Chem. Phys. 2018, 148, 174303 10.1063/1.5028393. 29739220

[ref80] Daxner M.; Denifl S.; Scheier P.; Echt O. Doubly charged CO_2_ clusters formed by ionization of doped helium nanodroplets. Int. J. Mass Spectrom. 2014, 365, 200–205. 10.1016/j.ijms.2014.01.016. 25844051 PMC4375666

[ref81] Mauracher A.; Daxner M.; Huber S. E.; Postler J.; Renzler M.; Denifl S.; Scheier P.; Ellis A. M. Formation of Dianions in Helium Nanodroplets. Angew. Chem., Int. Ed. 2014, 53, 13794–13797. 10.1002/anie.201408172. 25296629

[ref82] Renzler M.; Harnisch M.; Daxner M.; Kranabetter L.; Kuhn M.; Scheier P.; Echt O. Fission of multiply charged alkali clusters in helium droplets - approaching the Rayleigh limit. Phys. Chem. Chem. Phys. 2016, 18, 10623–10629. 10.1039/C6CP00764C. 27035406

[ref83] Denifl S.; Zappa F.; Mähr I.; Mauracher A.; Probst M.; Urban J.; Mach P.; Bacher A.; Bohme D. K.; Echt O.; et al. Ionization of doped helium nanodroplets: Complexes of C_60_ with water clusters. J. Chem. Phys. 2010, 132, 234307 10.1063/1.3436721. 20572705

[ref84] Saito Y.; Ishida T.; Noda T. Cluster ions ejected from an Li-Mg alloy liquid metal ion source: Observation of Mg_2_^2+^ and Mg_3_^2+^. J. Am. Soc. Mass Spectrom. 1991, 2, 76–80. 10.1016/1044-0305(91)80063-D. 24242091

[ref85] Hogreve H. Mg_2_^2+^: a long-lived metastable dication. Chem. Phys. Lett. 2004, 394, 32–36. 10.1016/j.cplett.2004.06.099.

[ref86] Bogdanov E.; Demidov V. I.; Kaganovich I. D.; Koepke M. E.; Kudryavtsev A. A. Modeling a short dc discharge with thermionic cathode and auxiliary anode. Phys. Plasmas 2013, 20, 101605 10.1063/1.4823464.

[ref87] Jellinek J.; Acioli P. H. Magnesium clusters: Structural and electronic properties and the size-induced nonmetal-to-metal transition. J. Phys. Chem. A 2002, 106, 10919–10925. 10.1021/jp020887g.

[ref88] Acioli P. H.; Jellinek J. Electron binding energies of anionic magnesium clusters and the nonmetal-to-metal transition. Phys. Rev. Lett. 2002, 89, 213402 10.1103/PhysRevLett.89.213402. 12443408

[ref89] Li K. C.; Stwalley W. C. Vibrational levels near dissociation in Mg_2_ and long-range forces. J. Chem. Phys. 1973, 59, 4423–4427. 10.1063/1.1680641.

[ref90] Köhn A.; Weigend F.; Ahlrichs R. Theoretical study on clusters of magnesium. Phys. Chem. Chem. Phys. 2001, 3, 711–719. 10.1039/b007869g.

[ref91] Kittel C. Introduction to Solid State Physics, 8th ed.; John Wiley & Sons, Inc: Hoboken, NJ, 2005.

[ref92] Zöttl S.; Kaiser A.; Daxner M.; Goulart M.; Mauracher A.; Probst M.; Hagelberg F.; Denifl S.; Scheier P.; Echt O. Ordered phases of ethylene adsorbed on charged fullerenes and their aggregates. Carbon 2014, 69, 206–220. 10.1016/j.carbon.2013.12.017. 25843960 PMC4375791

