# Supplementary material for: Mixed Cluster Ions of Magnesium and C60
Source: J Phys Chem A. 2024 Jan 25;128(5):848–57. doi: 10.1021/acs.jpca.3c06902 (PMC10860146; doi:10.1021/acs.jpca.3c06902)
Supplement: Supplementary file 1 — jp3c06902_si_001.pdf [file jp3c06902_si_001.pdf]

Supporting Information for Publication

Mixed Cluster Ions of Magnesium and C_{60}

Anna Maria Reider[1], Jan Mayerhofer[1], Paul Martini[1,2], Paul Scheier[1] and Olga V. Lushchikova[1,]*

[1]Institut für Ionenphysik und Angewandte Physik, Universität Innsbruck, Technikerstr. 25, A-6020 Innsbruck, Austria; Anna-Maria.Reider@uibk.ac.at

[2]Department of Physics, Stockholm University, 106 91 Stockholm, Sweden; paul.martini@fysik.su.se

[*]Correspondence: Olga.Lushchikova@uibk.ac.at

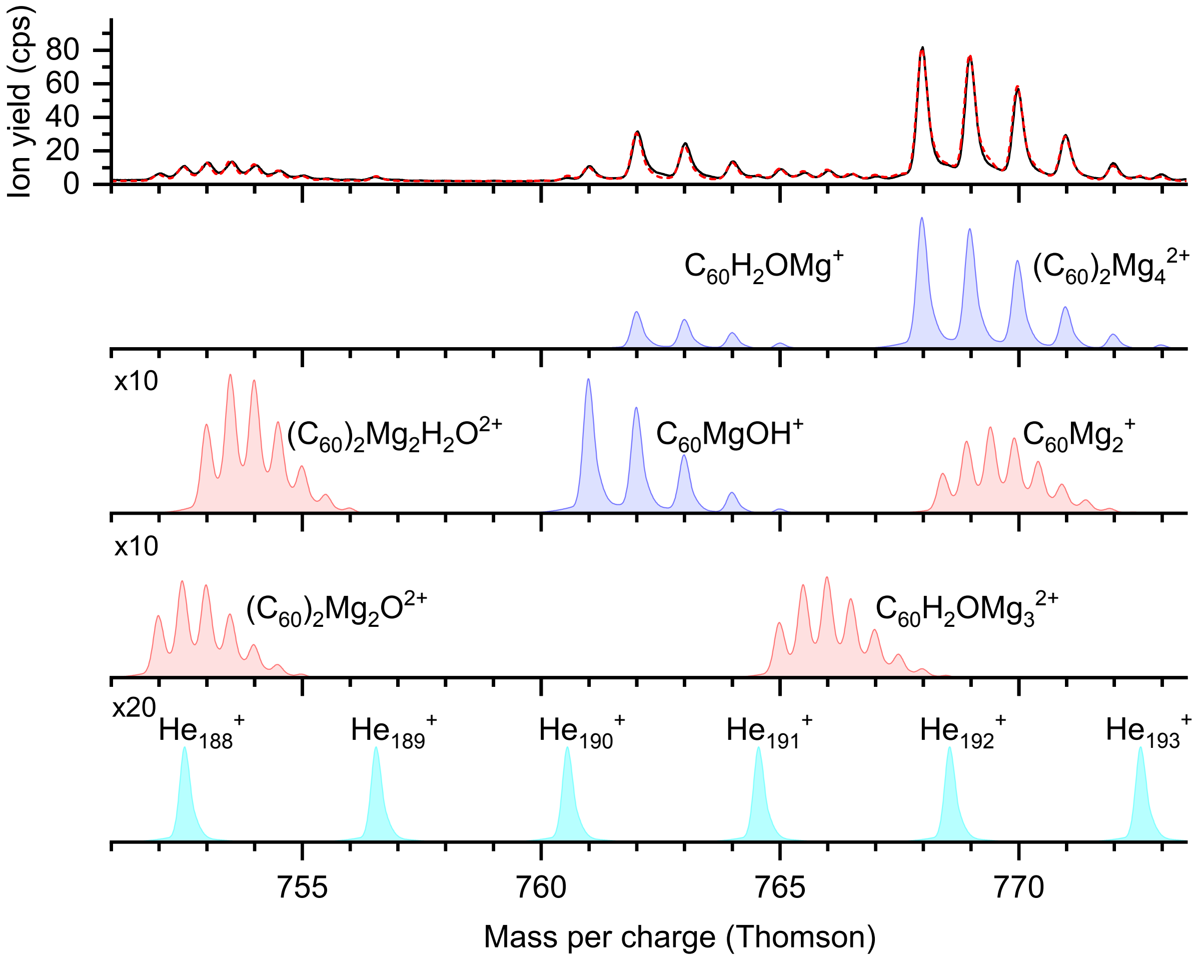

Figure S1: The upper diagram shows a section of the mass spectrum (solid black line) and the resulting fit from IsotopeFit (red dashed line). The isotopic patterns of all ions contributing to this fit are shown in the lower four diagrams.

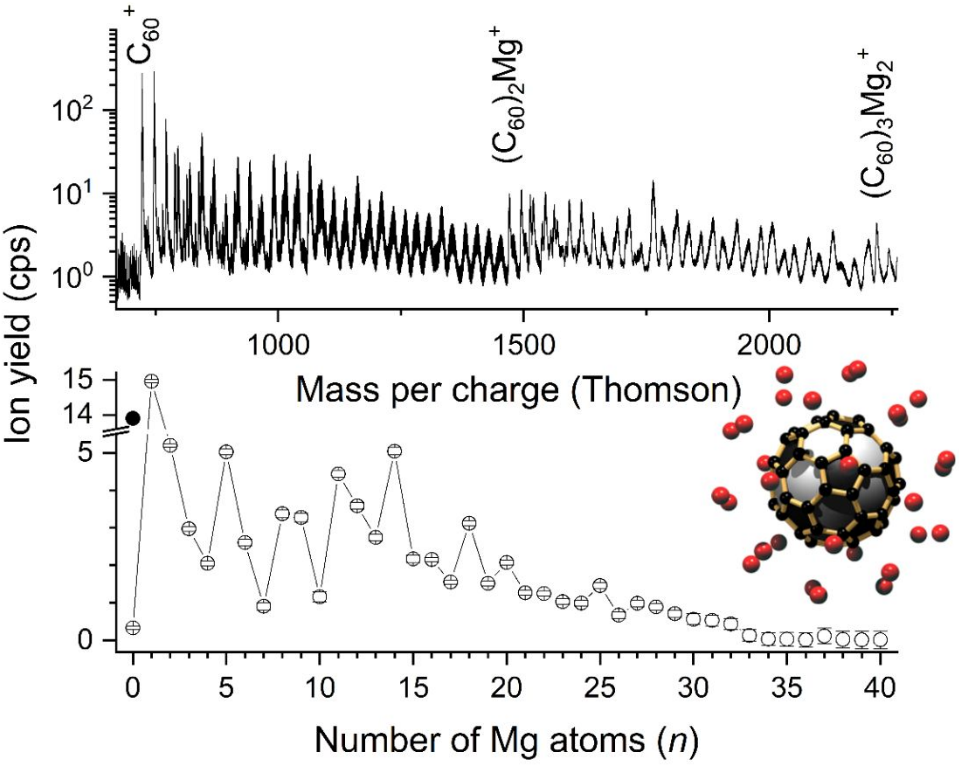

Figure S2: upper diagram: Mass spectrum of low-mass ions ejected from C_{60}/Mg doped helium nanodroplets upon multiple electron ionization. Parameters: He source: 2 MPa, 9.55 K, electron gun: 70 eV, 122 µA, C_{60} oven: 516 K, Mg oven: 648 K. Lower diagram: ion yield of $C_{60}Mg_n^+$ as a function of the number of magnesium atoms n extracted from the mass spectrum above utilizing the software IsotopeFit [1]. Please note the clear drop of intensity at $n = 32$ indicating the closure of a shell where one Mg atom occupies every pentagonal and hexagonal ring of the fullerene cage. Such a commensurate 1×1 decoration has been observed for larger alkaline earth metals [2] as well as for various light molecules and He adsorbed to C_{60}^+ [3-7] and C_{60}^- [8]. The inset in the lower diagram schematically shows the structure of $C_{60}Mg_{32}^+$.

1. Ralser, S.; Postler, J.; Harnisch, M.; Ellis, A. M.; Scheier, P., Extracting cluster distributions from mass spectra: IsotopeFit. *International Journal of Mass Spectrometry* **2015,** *379*, 194-199.

2. Zimmermann, U.; Malinowski, N.; Näher, U.; Frank, S.; Martin, T. P., Multilayer metal coverage of fullerene molecules. *Physical Review Letters* **1994,** *72*, 3542-3545.

3. Leidlmair, C.; Bartl, P.; Schöbel, H.; Denifl, S.; Probst, M.; Scheier, P.; Echt, O., On the Possible Presence of Weakly Bound Fullerene-H_2 Complexes in the Interstellar Medium. *Astrophysical Journal Letters* **2011,** *738*, L4.

4. Zöttl, S.; Kaiser, A.; Bartl, P.; Leidlmair, C.; Mauracher, A.; Probst, M.; Denifl, S.; Echt, O.; Scheier, P., Methane Adsorption on Graphitic Nanostructures: Every Molecule Counts. *Journal of Physical Chemistry Letters* **2012,** *3*, 2598-2603.

5. Echt, O.; Kaiser, A.; Zöttl, S.; Mauracher, A.; Denifl, S.; Scheier, P., Adsorption of Polar and Nonpolar Molecules on Isolated Cationic C_{60}, C_{70}, and Their Aggregates. *Chempluschem* **2013,** *78*, 910-920.

6. Zöttl, S.; Kaiser, A.; Daxner, M.; Goulart, M.; Mauracher, A.; Probst, M.; Hagelberg, F.; Denifl, S.; Scheier, P.; Echt, O., Ordered phases of ethylene adsorbed on charged fullerenes and their aggregates. *Carbon* **2014,** *69*, 206-220.

7. Leidlmair, C.; Wang, Y.; Bartl, P.; Schöbel, H.; Denifl, S.; Probst, M.; Alcami, M.; Martin, F.; Zettergren, H.; Hansen, K.*, et al.*, Structures, Energetics, and Dynamics of Helium Adsorbed on Isolated Fullerene Ions. *Physical Review Letters* **2012,** *108*, 076101.

8. Harnisch, M.; Weinberger, N.; Denifl, S.; Scheier, P.; Echt, O., Adsorption of helium on isolated C_{60} and C_{70} anions. *Molecular Physics* **2015,** *113*, 2191-2196.
